# Carbon Sequestration by Fruit Trees - Chinese Apple Orchards as an Example

**DOI:** 10.1371/journal.pone.0038883

**Published:** 2012-06-15

**Authors:** Ting Wu, Yi Wang, Changjiang Yu, Rawee Chiarawipa, Xinzhong Zhang, Zhenhai Han, Lianhai Wu

**Affiliations:** 1 College of Agronomy and Biotechnology, China Agricultural University, Beijing, China; 2 Rothamsted Research, North Wyke, Okehampton, United Kingdom; University of Illinois, United States of America

## Abstract

Apple production systems are an important component in the Chinese agricultural sector with 1.99 million ha plantation. The orchards in China could play an important role in the carbon (C) cycle of terrestrial ecosystems and contribute to C sequestration. The carbon sequestration capability in apple orchards was analyzed through identifying a set of potential assessment factors and their weighting factors determined by a field model study and literature. The dynamics of the net C sink in apple orchards in China was estimated based on the apple orchard inventory data from 1990s and the capability analysis. The field study showed that the trees reached the peak of C sequestration capability when they were 18 years old, and then the capability began to decline with age. Carbon emission derived from management practices would not be compensated through C storage in apple trees before reaching the mature stage. The net C sink in apple orchards in China ranged from 14 to 32 Tg C, and C storage in biomass from 230 to 475 Tg C between 1990 and 2010. The estimated net C sequestration in Chinese apple orchards from 1990 to 2010 was equal to 4.5% of the total net C sink in the terrestrial ecosystems in China. Therefore, apple production systems can be potentially considered as C sinks excluding the energy associated with fruit production in addition to provide fruits.

## Introduction

The contribution of orchards to carbon (C) cycling ranging from C storage [1], [2], root respiration [3], [4], [5], [6], [7] and net CO_2_ flux [8] has been published. Measured and simulated components of the C balance in apple trees have also been reported [9], [10]. However, there are very few studies that consider the impacts of orchard management practices on the environment and C cycle using a systems approach. The potential for C credits based on standing biomass for orchards is limited compared to forest stands growing in the same climatic zone. It is roughly estimated that New Zealand orchards could sequester less than 70 t C ha^−1^ within their lifespan of at least 25 years, while forest stands can sequester much more, e.g. *Pinus radiata*, reaching 300–500 t C ha^−1^ under New Zealand conditions [11]. However, orchards and forests may sequester similar amounts of C in the first few years after their establishment [11]. Although the comparison of C sequestration between forests and orchards has been made, the results were not produced based on the same criteria, e.g. living biomass was only considered without including indirect C emissions associated with orchard management practices. The content of soil organic matter depends largely on the periodic input of organic materials and the decomposition rate of soil organic matter [12]. Any increase of the soil C pool is the result of biotic C inputs that comes from CO_2_ fixation directly or indirectly by plants in agroecosystems. The fixed C is partitioned to different organs of the fruit trees, which depends on number of factors, e.g. genotype [13], tree age [14], orchard density [15], fruit production [16], training systems [17] and orchard management [18]. Meanwhile, it would be very difficult to quantify some components of C balance in an orchard system, e.g. the natural fall of flowers and fruits, microbial respiration and rhizodeposition in the overall C balance of an orchard [8]. Some of the C allocated to roots will return to the air as root respiration and enter the soils as rhizodeposits. The amount of C losses through these channels varies. It was reported that one quarter to one third of respiration occurring in a soil is from roots of higher plants, and rhizodeposition accounts for 2–30% of total dry matter production in young plants [19]. In contrast, C immobilised in short-life organs, like fruits and leaves, has other pathways [8]. Normally, C from fruits is removed from the orchard system through harvesting while that from leaves is translocated into the woody perennial parts of the apple trees or converted to soil organic C through decomposition after the leaves become litterfall.

China is the largest apple producer in the world with c.a. 35×10^6^t in the 2011/2 season (USDA). Fruit trees are an important component in the Chinese agricultural sector with 8.67 million ha of orchards, of which apple orchards cover 1.99 million ha [20]. Considering its storage capacity of fixed C and the size of planting area, the orchards in China could play an important role in the C cycle of terrestrial ecosystems and contribute to C sequestration. However, there is little knowledge on how to improve the C sequestration potential in Chinese orchards.

To accurately estimate C stocks and fluxes in orchards located in different regions, it is desirable to set a baseline that can eliminate the uncertainties caused by the variations in fruit tree structure, stocks and fluxes among and within geographical sites combined with direct field measurements from local sample plots. In natural and semi-natural forests, the C carrying capacity can be used as an indicator for the baseline [21], [22]. The indicator is made up of a matrix of factors to quantify forest degradation and sequestration in terms of C losses and gains due to land-use changes [21], [23], [24], [25]. Because orchards are managed systems, the estimation of C sequestration capacity is different from the natural forest. Therefore, it is necessary to build a new matrix for orchard ecosystems to accommodate management factors. The aim of this paper is 1) to set up an assessment matrix of C sequestration capability based on measured data to evaluate the C sequestration capability of apple trees of a range of ages; and 2) to assess the status of C sequestration by apple orchards in China.

## Materials and Methods

### Study Site

A field-modelling study was conducted in the apple orchards located in Changping District, Beijing (116°28′55′′E, 40°20′34′′N) between 2009 and 2010. The climate of this region is temperate continental monsoon with an annual average temperature of 12.1°C. The soil of the orchards is deep sandy loam or loam with approximately 1.2% of soil organic matter content in the 20 cm topsoil. Three orchards of Fuji/Makino apple trees, 5, 18 and 22 years old, were chosen (i.e. apple trees were planted in 2005, 1992 and 1988 separately), to represent juvenile, mature and over-mature phases in the life cycle of apple trees. Information on fertilizer use and irrigation application in the orchards was obtained via the Beijing Agricultural Technical Extension Station. General information about the orchards is shown in Table 1. Four frames [26] in each orchard were randomly set up but avoiding the influence of diseases and insects on the trees within the frames to determine the value of stem diameter. The value with the highest probability within the orchard using the normal distribution of the stem diameters was taken to identify an apple tree to be harvested for biomass estimation. Three frames from the four quadrats in the orchard were chosen for fine-root observations with minirhizotron, soil core samplings for fine-roots and soil respiration measurements. The area of the quadrat was 2×4 m^2^ for the 5-year-old orchard and 3×5 m^2^ for the 18- and 22-year-old orchards, considering the planting densities of the orchards with different ages.

**Table 1:** General information of the Fuji apple tree orchards at Changping, Beijing.

	5-yr-old	18-yr-old	22-yr-old
Orchard size (ha)	0.33	1.67	0.33
Nitrogen application (kg N ha^−1^ yr^−1^)	135	149	149
Phosphorus application(kg P ha^−1^ yr^−1^)	135	149	149
Potassium (kg P ha^−1^ yr^−1^)	135	149	149
Animal manure (kg ha^−1^ yr^−1^)*	87000	99000	97500
KNO_3_ (kg ha^−1^ yr^−1^)	300	330	330
ZnSO_4_ (kg ha^−1^ yr^−1^)	300	330	330
Bacterial fertilizer (kg ha^−1^ yr^−1^)	300	330	330
Irrigation amount (m^3^ ha^−1^ yr^−1^)	3220	3300	3300

* OM content: about 25%; nutrient content: N - 1.6%, P - 1.5% and K - 0.8%.

### Analysis of Carbon Sequestration Capability

Matrix analysis was used to investigate the capability of C sequestration. Three indirect sources for C emission: irrigation, and chemical fertilizer and manure applications were identified. A set of the potential assessment factors, *U_i_* (*i* = 1,2,3, representing the 5-, 18- and 22-year-old orchards, respectively), was formed with the subset in the following order: long residence woody (stem + branch + coarse root), leaf, fruit, fine root, pruning, soil respiration, irrigation and fertilizer application. Because the pruned branches would normally leave the orchard systems immediately, this component is treated as a source of C emissions within the orchards in this study. There are two columns in *U_i_*: the C capture elements (*U_i1_*) and the C emission elements (*U_i2_*), which were used to evaluate the contribution of the assessment factors to C sequestration in an orchard. The C capture by long residence of woody, leaf or fruit was represented with the C content of increased biomass. The contribution to C emissions from fertilizer or irrigation is calculated by amount of fertilizer application or irrigation multiplied by a conversion coefficient which was adopted from *West and Marland* [27].

A vector (*B_i_*) was used to assign weighting factors for each subset in *U_i_*. The values in the vector reflect the contribution of the factors in the overall assessment for orchard *i*. The elements in *B_i_* include: weighting factors of long residence woody, leaf, fruit, fine root, pruning, soil respiration, irrigation and fertilizer application in order. The matrix *V_i_* derived from the product of the matrix and its corresponding vector was the outcome of the C capture and emission from the orchard *i*:
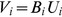
(1)


The measurement of the potential assessment factors described were as below.

### Biomass Estimation

Three representative apple trees from each orchard were identified in September, 2009 and the increments in stem diameter at 20 cm from the ground of the trees were measured in October, 2009 to October, 2010. Increment of stem diameter measurements one year apart were used to estimate the biomass increment for the experimental year. The trees were divided into leaves, branches, main stems and coarse roots, and all fresh weights were measured in the field. Then sub-samples from each fraction were dried to determine total dry matter of each fraction. The sub-samples were oven-dried at 65°C until constant mass was reached. The dried sub-samples were ground after weighting before total C analysis was made by EA 1108 elemental analyser (Italy, Carlo Erba) to determine C content. The data were used to fit allometric equations to quantify the relationship between the biomass of different parts (leaf, stem, branch, coarse root) and stem diameter. Because of the life cycle of an apple tree, fruit production would begin to decline in the over-mature phase year by year. Therefore, a parabola equation was used to quantify the relationship between fruit production and stem diameter. Statistical analysis was made using SPSS for Windows (Rel. 11.5.0, 2002. Tokyo: SPSS Inc.).

**Figure 1:**
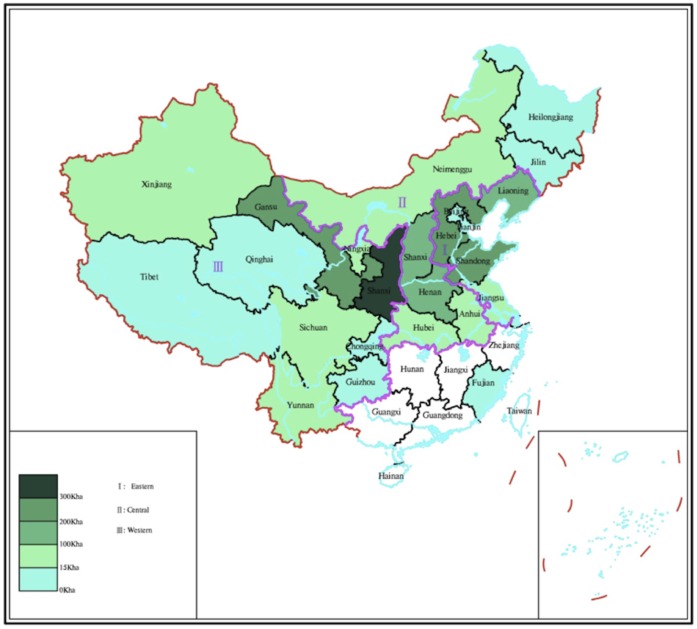
Distribution of apple grown area in China. The regions without colour indicate non-apple grown areas.

**Figure 2:**
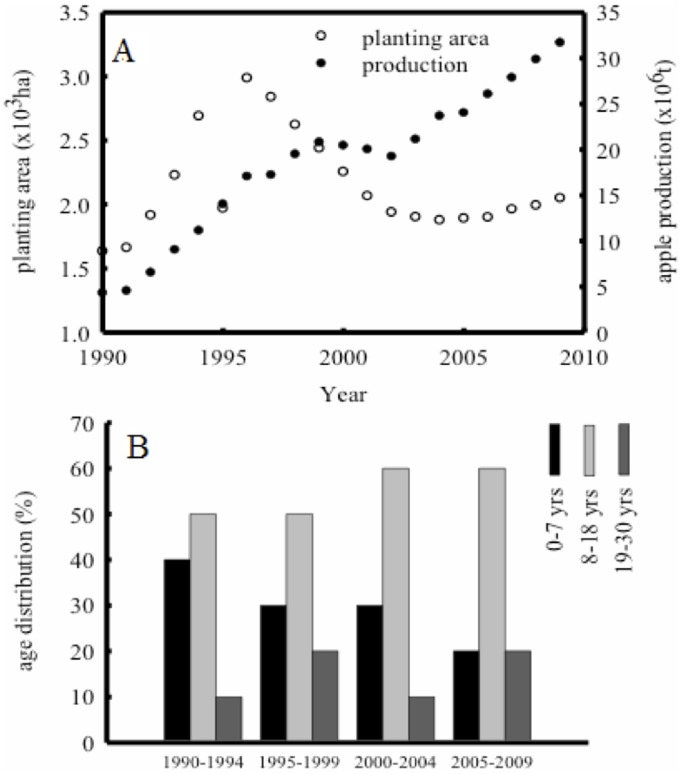
Dynamics of apple orchards area and production (A) and age distribution of the orchards (B) between 1990 and 2010 in China [*data source: Editorial Committee*, 1991;1996;2001;2006;2010].

### Fine Roots Observation

The turnover rate of fine roots is an important parameter to indicate the contribution of the fine roots to C sequestration. Minirhizotron and soil coring method were used to estimate the turnover rate.

In July, 2009 before the experiment started, nine 90 cm-long minirhizotron tubes of 5 cm diameter were inserted into the soil at 45° angle with the horizon in the second quadrat of each orchard to allow fine roots to settle in the soil surrounding the tubes. Minirhizotron images were collected every ten days using a BTC-10 minirhizotron microscope (Bartz Technology, USA) from October 2009 to October 2010. This generated 64 images from each minirhizotron tube. The length of fine roots was calculated for all the images using the I-CAP software. The collected information was used to calculate the length of fine roots (cm) per unit area. A fine root turnover index, defined as the ratio of fine root mortality in a year (cm cm-1) to initial fine root length (cm cm^−1^) within the minirhizotron window [26], was calculated using the data with the reported method. Within a quadrat from each orchard, ten soil cores of 4 cm in diameter and 80 cm in depth were sampled. The soil columns were separated into depths 0–20, 20–40, 40–60 and 60–80 cm. The soil samples were transferred in plastic bags. Roots were manually picked out from the samples, washed and sorted (<2 mm), then they were oven-dried at 80°C until constant mass were reached. Fine root dry matter density (mg DM cm^−3^) was estimated based on the relationship between the weight and the surface area of sampled fine roots, which was used to estimate the biomass of fine roots.

### Soil Respiration

Soil respiration (and temperature) at 10 cm soil depth in the three quadrats in each orchards were measured every ten days between June 2009 and June 2010 (excluding first three months in 2010 when the top soil was frozen) using developed closed gas-exchange system (LiCor 6400 Portable Photosynthesis System with 6400-09 soil CO_2_ flux chamber; LiCor, Lincoln, NE, USA). At all three orchards, 27 replicate LiCor soil collars in total were installed. The collars remained in place throughout the experiment period, allowing repeated measurements. All measurements were carried out between 10:00 to 11:30 am. Measurements were not made on days of rain.

**Table 2:** Allometric equations for dry matter (kg) for different parts of subject trees using the square of the stem diameter (cm) at 20 cm from the ground as an independent variable (sample number is 9 for each component).

	Formula	*a*	*B*	*c*	Adjusted R^2^
Fruit	*a*x^2^+*b*x+*c*	−1.170	0.260	−0.001	0.809
Branch	*a*x*^b^*	0.124	1.234		0.984
Stem		0.178	1.101		0.997
Leaf		0.160	0.656		0.951
Root		0.159	0.999		0.946

### Litterfall Decomposition

At the beginning of September 2010, 20 g fresh leaves collected from the apple trees was put into a nylon bag with a mesh size of 2 mm. Six bags were randomly placed on the surface of the orchard soil (after removing the litter layer). At the end of each month for the following three months, two litterbags were collected from each orchard to calculate weight loss and C content of litterfall. The data was used to determine decomposition rate by fitting the exponential function [28]:
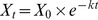
(2)where *X_0_* is initial C content of the litterfall (g C), *X_t_* is C content at time *t* and *k* is the decomposition rate (d^−1^). Data processing and allometric equations.

### Other Data Sources

There are three geographical regions for apple production in China: the western, the central and the eastern regions. This study covered major apple regions in China (Figure 1), biomass data of apple trees at various ages from geographic locations was used to validate the allometric equations described in 2.2. The dynamics of the net C sink and C storage of apple orchards in China was calculated based on cultivation areas and tree age groups (Figure 2). The groups were set 0–7, 8–18 and 19–30 years old at national level.

**Table 3:** Biomass (kg tree^−1^) of various parts of an apple tree with various ages in different geographical areas in China.

Site	Age (yr)	Stem diameter (cm)	Fruit	Branch	Stem	Coarse root	Leaf	Source*
North China	5	3.19	0.98	1.44	2.23	3.21	0.70	1
		3.11	1.58	2.25	2.38	1.10	0.76	1
		3.42	1.64	3.18	2.32	1.19	0.76	1
	18	13.22	26.06	83.29	52.47	23.84	5.73	1
		12.02	17.31	68.02	40.64	30.77	3.61	1
		13.42	14.60	92.00	52.21	29.48	7.11	1
	22	13.69	12.76	67.44	61.39	22.93	3.574	1
East China	7	5.55	2.42	7.82	7.10	4.91	1.62	2, 3
	8	5.51	9.74	5.22	24.24	12.19	3.47	2, 4
		5.51	6.52	5.81	20.20	7.71	4.35	5
	17	11.58	9.00	8.12	26.86	19.47	3.12	2, 6
		11.58	12.23	10.35	12.64	13.68	4.09	2, 5
Northwest China	5	3.58	0.54	1.73	1.69	0.94	0.55	7

Source*: 1 survey by the authors; 2–6 from per. comm. (2 Y. Jiang; 3 L. Zhao; 4 J. Fang; 5 D. Zhang; 6 N. Ding); 7 Zhang et al. [2009].

**Table 4:** Comparison of the modelled and observed total dry matter content of an apple tree (kg).

stem diameter range	0–4 cm	4–6 cm	10–15 cm
Modelled	7.5±1.0	16.9±0.2	154.1±31.9
Observed	6.6±1.2	29.2±12.1	138.0±57.6

**Figure 3:**
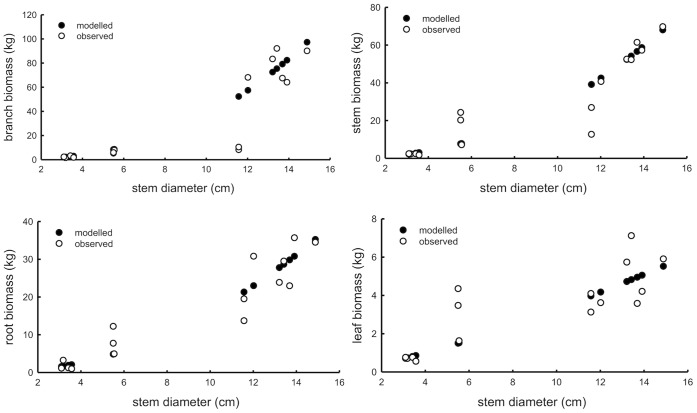
Comparison between the modelled and observed dry matter of branch, stem, fine root and leaf with different stem diameters.

**Figure 4:**
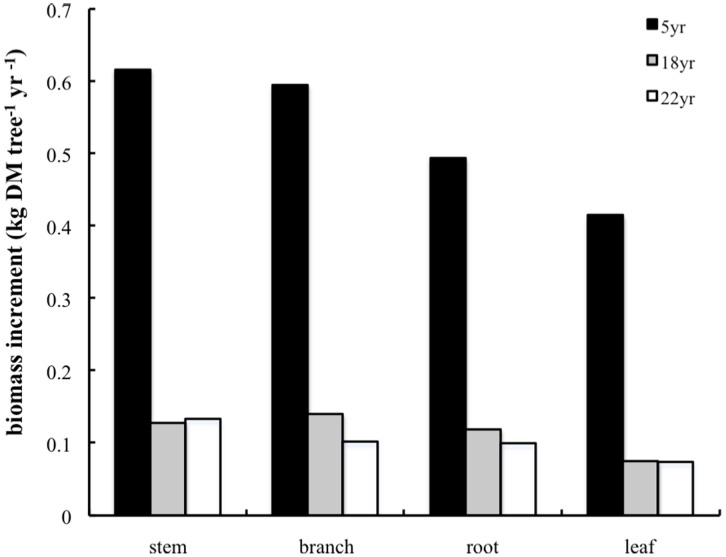
Annual biomass increment rates in various living parts of apple trees with different ages between Oct. 2009 and Oct. 2010.

## Results

### Carbon Storage, Emission and Turnover of Apple Trees

#### C capture of long residence woody, leaf, fruit

Carbon storage from a part of an apple tree is estimated based on dry matter and C content of the part. The average stem diameters at 20 cm from the ground were 3.2±0.2, 12.9±0.8 and 14.2±0.6 cm for the 5-, 18- and 22-year-old trees. The parameter values for the allometric equations to estimate dry matter from different parts of an apple tree are presented in Table 2.

**Figure 5:**
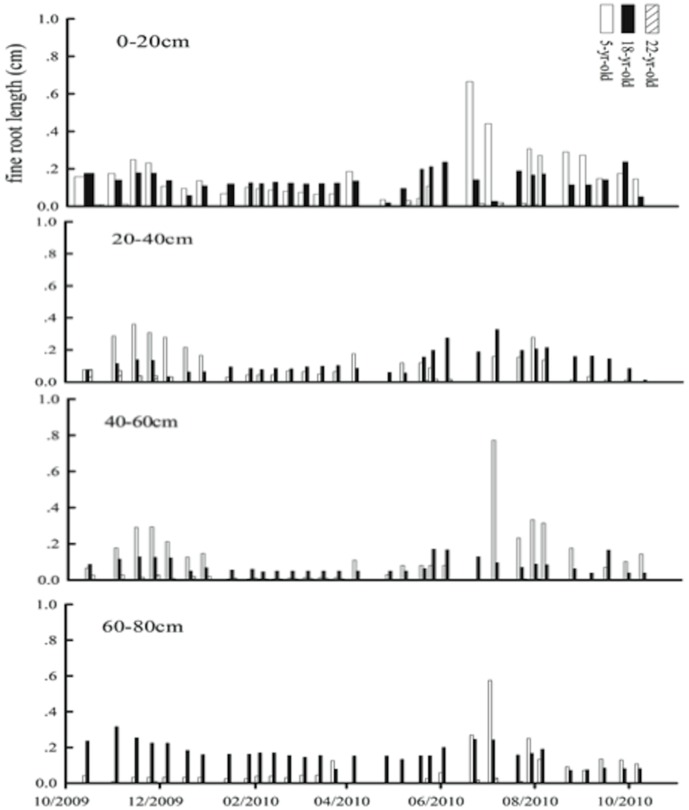
Dynamic changes of total fine root length in different soil layers in three orchards between Oct. 2009 and Oct. 2010.

**Figure 6:**
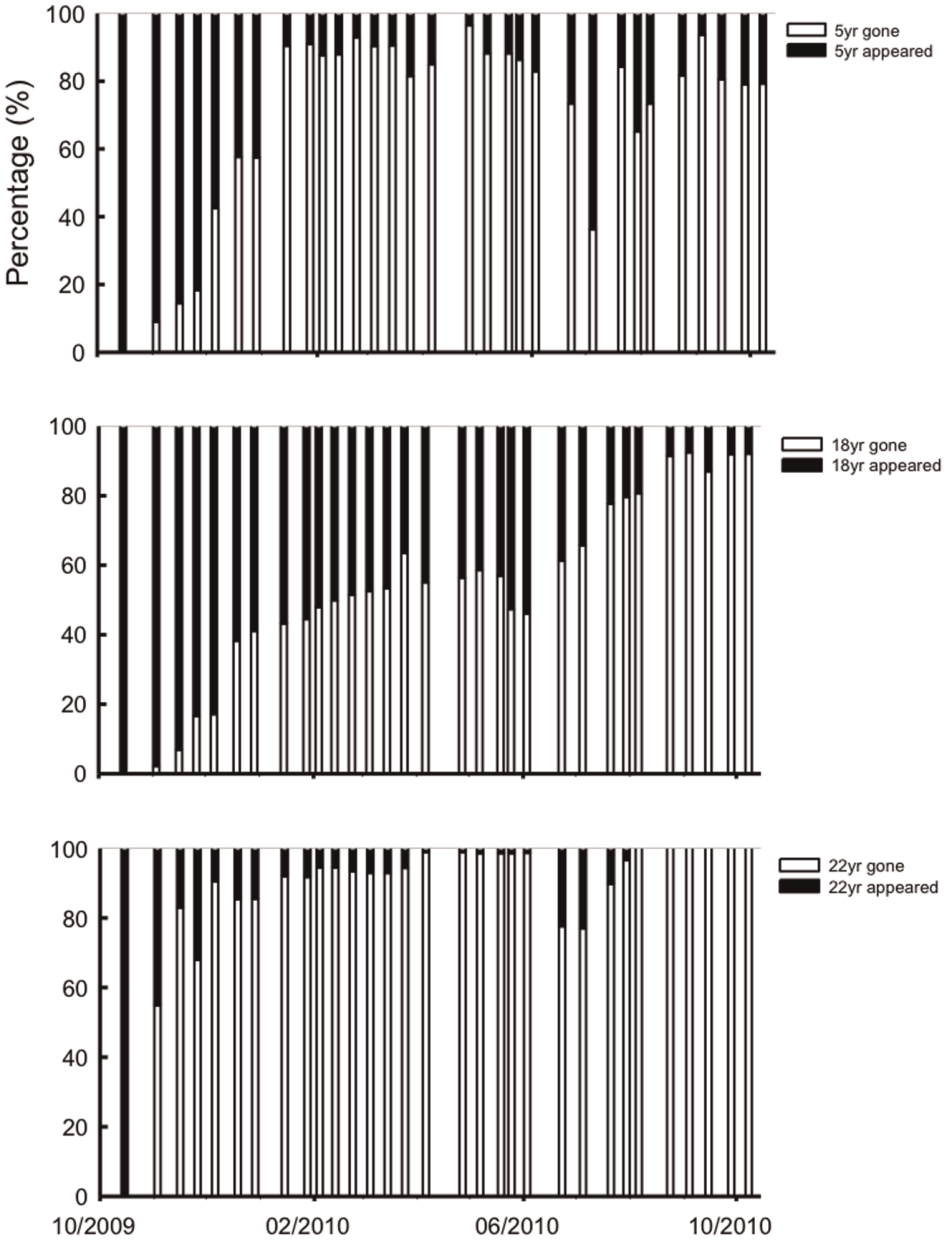
Dynamics of the proportions of living roots and disappeared roots within the minirhizotron window between Oct. 2009 and Oct. 2010.

**Table 5:** Dry matter and carbon content in pruned branches of an apple tree with different ages assuming 3.75 m^2^ of land is covered by an 18- or 22-year-old tree and 2 m^2^ for a 5-year-old tree.

tree age (yr)	5	18	22
Dry matter (kg tree^−1^)	0.587	3.457	4.630
C content (kg C tree^−1^)	0.270	1.590	2.130
C content (kg C m^−2^)	0.135	0.424	0.568

In order to validate the allometric equations which can be applied to the apple production regions in China, biomass data of an apple tree were collected dated from 1990 in major apple production regions (Table 3). The observed biomass and estimated values from the equations were compared (Table 4 and Figure 3). For the trees with stem diameter between 4 and 6 cm, the equations underestimated biomass of trees due to a large standard deviation in the collected data. For the other two groups at stem diameters of 0–4 cm and 10–15 cm, the estimated values confirm to the actual ones. These results indicated that the allometric equations should be suitable for biomass estimation of apple trees which were subjected to managed agricultural production systems.

The annual biomass increments of living organs of apple trees at different ages were calculated using the equations. The increments of an individual tree were converted into the increments per unit land area through multiplying tree density. The results showed that the 5-year-old tree has a much higher growth rate than the other two groups although its standing biomass is low (Figure 4). The growth rates of all parts (except stem) for the trees older than 18 years began to slow down, which may be caused by reduced growth of a mature tree. As fruit trees get older, the proportion of the long residence woody biomass in total standing biomass production also increases.

**Figure 7:**
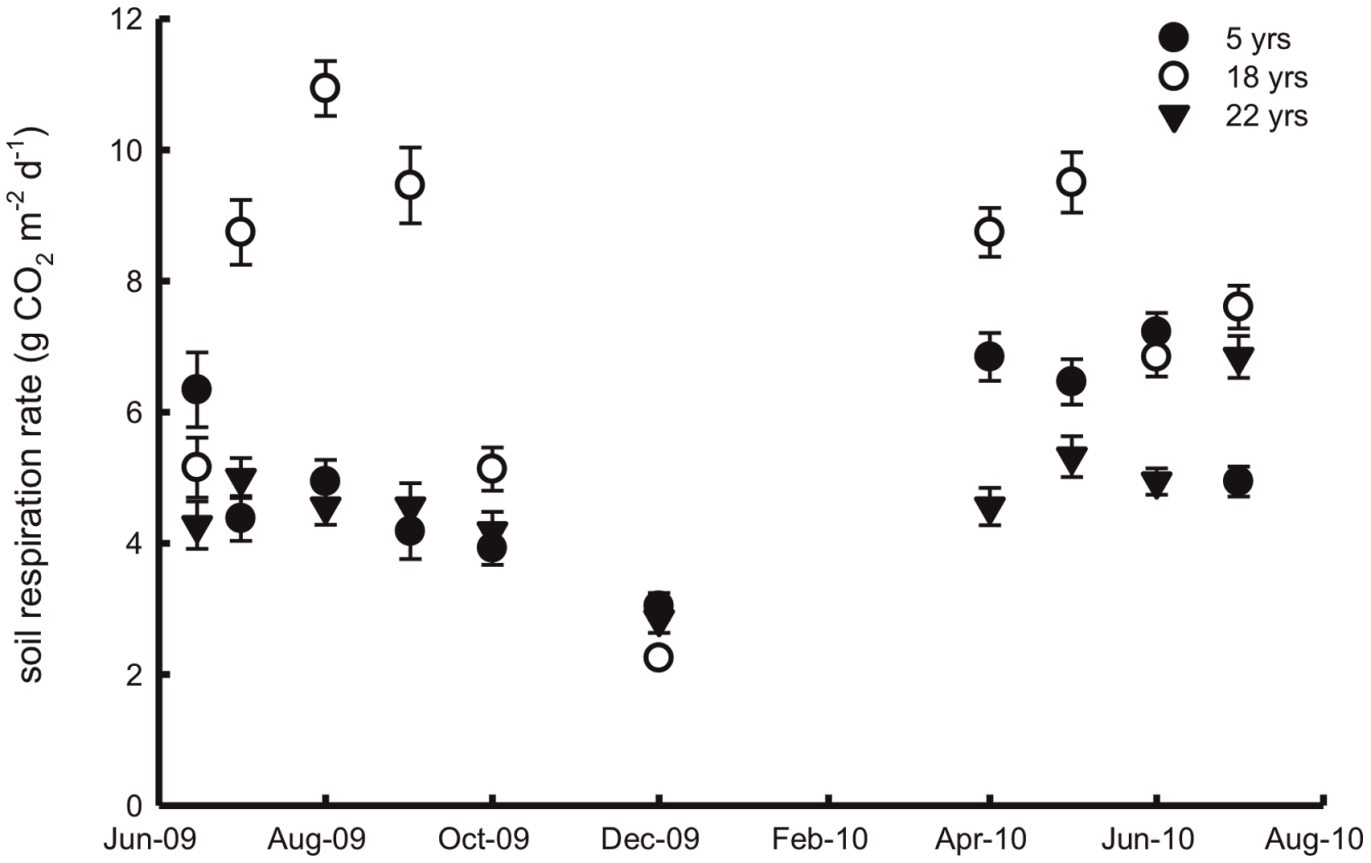
Soil respiration rates in the observed months in the 5-, 18- and 22-year-old apple orchards during the observation period.

**Table 6:** Carbon capture and emission (kg C m^−2^ yr^−1^) from the evaluation factors in the orchards with different planting ages.

		Long residencewoody	Leaf	Fruit	Fine root	Pruning	Soil respiration	Irrigation	Fertilizers
5 yrs old	Capture (*U_11_*)	0.47	0.091	0.17	0.015	0	0	0	0
	Emission (*U_12_*)	0	0.0026	0	0	0.135	1.25	0.017	0.034
18 yrs old	Capture (*U_21_*)	2.2	0.676	2.37	0.019	0	0	0	0
	Emission (*U_22_*)	0	0.192	0	0	0.424	1.59	0.017	0.038
21 yrs old	Capture (*U_31_*)	2.3	0.56	1.7	0.008	0	0	0	0
	Emission (*U_32_*)	0	0.159	0	0	0.568	1.21	0.017	0.038

#### C capture and turnover of fine roots

The analysis of minirhizotron images indicated that the net growth rate of fine roots had apparent seasonal changes for the trees of all the ages. Two growth peaks appeared, in early summer and late fall for the 5- and 18-year-old trees throughout the observed soil profile. However, no significant seasonal change in growth rates was observed in the 22-year-old trees. The active growing zone of fine roots fell between 20 and 60 cm of the soil profile (Figure 5). Based on the observation and the regression analysis between fine root weight and surface area (*W_fr_* = 0.634+0.7689*S_area_*, R^2^ = 0.8119 and n = 5), the annual growth rate of fine roots was calculated as 33.4±16.8, 41.7±19.0, and 17.7±6.8 g DM m^−2^ for the 5-, 18- and 22-year-old trees, respectively.

The indices of annual fine root turnover were 7.7, 6.8 and 1.5 for the 5-, 18- and 22-year-old trees, respectively. The proportion of appeared and disappeared fine roots in the minirhizotron window for the 5-year-old trees remained at a similar value for the longest period compared to those with other two ages (Figure 6), which indicated that the 5-year-old trees not only produced a large amount of fine roots, but their root systems also had a high metabolic rate.

**Table 7:** Percentages of various components in net carbon sequestration in the apple orchards with different ages.

Orchard age (yr)	5	18	22
Long residence woody (%)	22	36	45
Short-lived tissues (%)	12	29	18
Orchard managements (%)	66	35	37

#### C emissions of pruning and soil respiration

In China, each individual orchard follows its own guidelines for the disposal of pruned branches based on the age of trees. In general, more branches are pruned with tree ages. Biomass in pruned branches at different ages was shown in Table 5.

Soil respiration from the 18-year-old orchard showed the strongest seasonal changes among the orchards, and two peaks in a year existed (Figure 7). We observed that the highest rate for the 5-year-old orchard occurred in spring and early summer, and that for the 22-year-old orchard occurred in the middle of summer. Based on the samples, annual soil respiration rates were 1.3±0.3, 1.6±0.6 and 1.2±0.5 kg C m^−2^ in the 5-, 18- and 22-year-old orchards, respectively.

#### C emissions of litterfall

The data from the litterfall bags showed that the decomposition of litterfall followed an exponential equation:
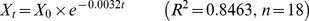
(3)


The total decomposition rate was equivalent to 312 days of turnover time. i.e., complete decomposition of litterfall would occur within a year either through emission to the atmosphere as CO_2_, or transformation into a stable organic matter pool in the soil.

### Carbon Sequestration Capability with Various Ages of Apple Trees

#### Contribution of the components to carbon sequestration

Annual C increment rate for each part of the trees in an orchard was treated as a C sink. The value of the assessment elements related to tree biomass was calculated by the allometric equations, plant density and the conversion fraction of dry matter to C content. The fraction was 0.46 g C g^−1^ DM based on the measurement of this study. In an annual cycle, leaves stay on trees during the growing season and then fall to the ground as litterfall for a certain period. During the year, some of litterfall decomposes releasing C to the atmosphere. Considering the length of the period and the measured decomposition rate, the emitted fraction of C in leaves is set at 0.284. Pruned branches, soil respiration and orchard managements are considered as sources of C emissions. The matrix of assessment elements for each age group of the orchard was shown in Table 6.

**Figure 8:**
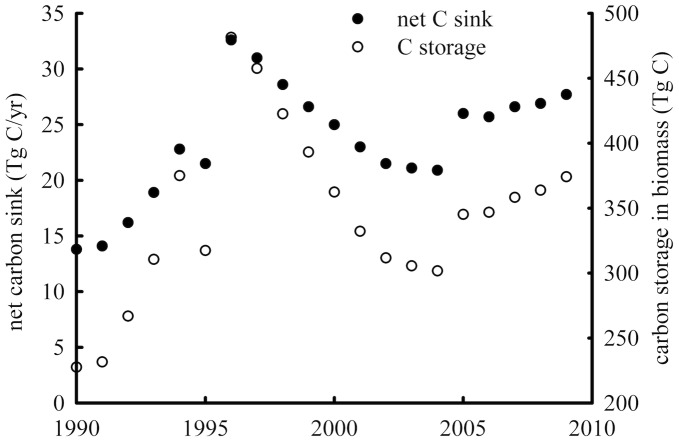
Net carbon sink in apple orchards in China between 1990 and 2010.

**Figure 9:**
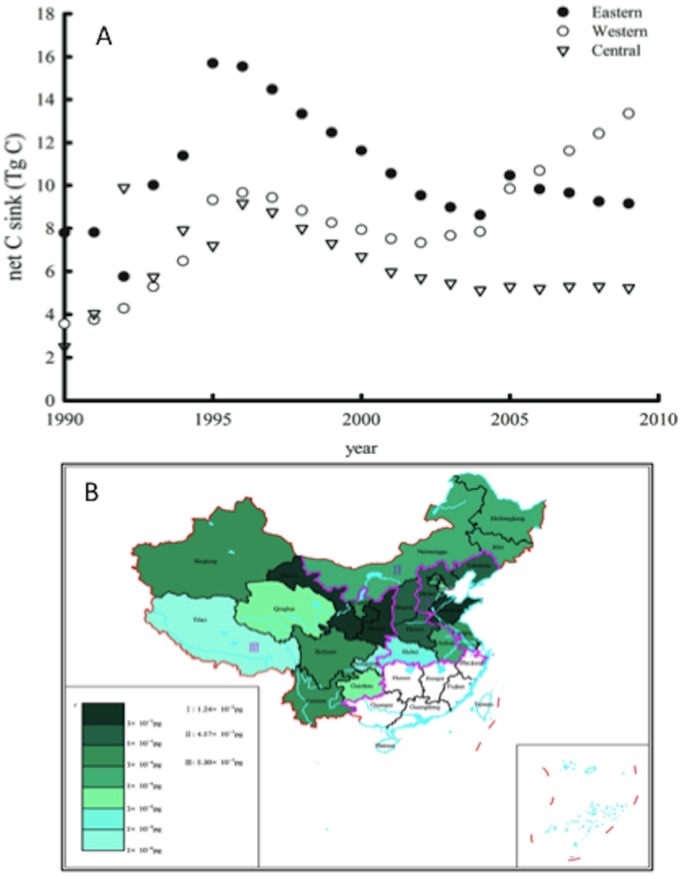
Dynamics of net carbon sink in apple orchards in the western, the central and the eastern regions between 1990 and 2010 (A), and the net carbon sink in each apple growing province in China in 2010 (B).

#### Weighting factors for the components

Because there are no substantial changes in long-term residence woody losses within one year, its weighting factor was set to 1. All leaves from apple trees will emerge at the beginning of the growing season and fall after the growing season of the year, so the weighting factor was also set to 1. An apple tree normally sets more fruits than they can be supported to grow and it usually becomes necessary to thin fruits or buds in order to improve the average size of each fruit remaining on the tree. Therefore, the fallen fruit or buds would be decomposed. Because harvested fruits usually grow for half a year, the weighting factor for harvested fruits in an orchard was given a score of 0.5. The annual fine root turnover index derived from the minirhizotron images is 7.69, 6.84 and 1.48 for the 5-, 18- and 22-year-old trees, respectively (details in 3.1.2). The orchard would be pruned once a year, its rate is also to be 1. C emissions from all types of orchard management practices were given a total score of 1. Therefore, the vector of weighting factors for the components from each orchard can be expressed as:
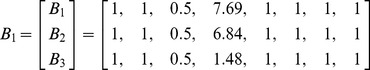
(4)


#### Assessment of carbon sequestration capability

The final evaluation matrix for each orchard (in a row) was produced through the product of *U* and *B*:
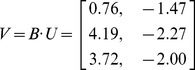
(5)


The first column in the matrix represents gross C input to an orchard and the second is C emission from the orchard. The net C sink by the end of the experimental period is −0.7, 1.9, and 1.7 kg C m^−2^ for the 5-, 18- and 22-year-old orchards, respectively. There is a transition point from net C emission to net C sink with the tree ages between 5 and 18 years old. The relationship between tree age (*X_age_*) and C sequestration (*C_seq_*) could be expressed with a parabola function:
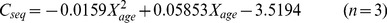
(6)


The equation showed that an eight-year-old orchard may reach the balance between the source and the sink. The apple trees older than 8 years could be considered as the C sink. When the trees are 18 years old, they reach the peak of C sequestration capability which then begins to decline with their ages.

The proportions of C stocks in short-lived tissues and long residence woody and C emissions from orchard management practices in C sequestration were analyzed (Table 7). In the 5-year-old orchard, the proportion of C emission from the orchard management practices far exceeds the portion of C stocks from both short-lived tissues and long residence woody. As the orchard enters the aging process, the proportion of short-lived tissues is declining, which could explain the lower C sequestration capability in the 22-year-old orchard.

### Carbon Sequestration Capability in Apple Orchards in China

There was a great development in apple tree production in the 1980s in China because of government incentives. As a consequence, most of seedling apple trees were planted then. Apple trees younger than 8 years old accounted for the largest percentage in the apple orchards by 1990. Therefore apple orchard inventory data from 1990s were analyzed in this paper. The evaluation matrix for the 5-, 18- and 22-year-old apple orchard generated from above session were used to estimate net C sink from 1990–2010 for the age group categories: 0–7 yr., 8–18 yr., and 19–30 yr., respectively. C storage in the living organs of apple trees at national level was assessed using the allometric equations. The estimated net C sink from apple orchards and the C storage of the apple trees in China were shown in Figure 8. It is noted that the C sequestration capability declined from 1995 to 2000. This is because the market price of apple decreased during the period, and a large number of tress were cut by farmers, especially in the eastern and northeast China. As a result, the total orchard area was reduced during this period.

The contribution from each region to total C sink from apple orchards is different and variable (Figure 9a). In the eastern and western regions, the change of net C sink from 2000 to 2005 had a similar trend as the national level shown in Figure 8. The sink in the eastern region has decreased, whilst it has kept rising in both the western and the central regions since 2005. The net C sink is 13.3, 5.24 and 9.15 Tg year^−1^ in the western, central and east regions in 2010, respectively. The estimation indicated that the apple orchards in the western region are the major contributor to the C sequestration (Figure 9b). The total net C sink from apple orchards in China is about 27 Tg C year^−1^ in 2010, which is equivalent to sequester 14 t C ha^−1^ year^−1^.

## Discussion

Monitoring the dynamics of fine roots and soil respiration, litterfall decomposition and destructive sampling on apple trees were made in three apple orchards with 5, 18 and 22 years old, respectively. The results showed that the 5-year-old tree had the highest growth rate among the trees with different ages and the rates of branches and roots for the trees >18 years began to slow down. The analysis of minirhizotron images indicated that the annual fine root turnover index is 7.69, 6.84 and 1.48 for the 5-, 18- and 22-year-old trees, respectively, but the 5-year-old tree produced a large amount of fine roots and its root system also had a high metabolic rate. Soil respiration from the orchard with 18 years old had the strongest seasonal changes among the orchards and two peaks in a year existed. Blanke (1996) [29]suggested Soil and grass respiration occupied a major contribution to the CO_2_ flux in a fruit orchard. Consistently our data show the annual respiration rates are 1.25, 1.59 and 1.21 kg C m^−2^ in the 5-, 18- and 22-year-old orchards, respectively. That means there’s no significantly relationship between apple trees and respiration. Soil respiration in 18 yr orchard is higher than the respiration in the other orchards (5-, 22-year-old orchards). We suppose that more animal manure applied to 18–year-old orchard (Table 1) which accelerate the soil respiration. The litterbag experiment made a conclusion that the residence time for litterfall is about 312 days. It is noted that the conclusion was drawn only from the results of a single year experiment. Ideally the experiment should last longer in order to acquire more data for the analysis of C sequestration capability. But it is a time-consuming, labour intensive and expensive experiment. It would be difficult to purely rely on field experiment for the analysis. Modelling may be an option to estimate the contribution of each component in an apple orchard system to C sequestration based on the obtained results from this experiment.

The results of C sequestration capability matrix suggested that the net C sink is −0.7 (source), 1.9, and 1.7 kg C m^−2^ for the 5-, 18- and 22-year-old orchards, respectively. The apple trees older than 8 years could be considered as a C sink. When the trees are 18 years old, they reach the peak of C sequestration capability which then begins to decline with their ages. Only when apple trees grew till a certain age in an orchard, the orchard could start to sequester C. Carbon emission derived from management practices would not be compensated through C storage in apple trees before reaching the mature stage. After an orchard became a net C sink, short-lived tissues turnover rates would be the major factor affecting C sequestration in the orchard. Based on apple orchard inventory data from 1990s and the evaluation matrix results from modelled plot in Beijing, it was estimated that the net C sink in apple orchards could range from 13.8 to 32.6 Tg C and the C storage of the apple trees from 227 to 481 Tg C during the period of 1990–2010. Among the three major growing regions of apple trees in China, the apple orchards in the western region are the major contributor to the C sequestration. The total net C sink from apple orchards in China is about 27 Tg C in 2010, which is equivalent to 14 t C ha^−1^. If taking the life cycling of apple trees as 25 years, the figure is similar to the reported amount for organic kiwifruit and apple production systems in New Zealand [30]. It is predicted that the capability continues growing substantially in China in the future because of the increase of growing areas and more apple orchards entering the mature stage. Therefore apple production systems can be potentially considered as a C sink apart from the function of fruit production.

Net ecosystem productivity is often used as a parameter for C sink at a regional level [11], [31] without considering the impact of orchard management practices. In this study, indirect C emissions from fertilizer applications and irrigations, as well as the direct contribution from soil respiration and tree pruning were included in the estimation of the capability, in addition to the biomass turnover rates. The results suggested that a young orchard would be a source of C emissions initially, and then it become a C sink from the eighth year when the trees are at the full fruit stage and the capacity of C sequestration gradually increases until it reaches 18 years old. During the full fruit stage, apple trees are more active in photosynthesis and metabolism, which would have more C deposited into their organs. Meanwhile, C emissions from orchard management practices would remain at a constant level. As a result, net C sequestration capability could increase when an orchard is between the ages of 8 and 18 years old. In practice, fruit trees are often forced to enter the full fruit stage as early as possible in China. To achieve this goal, heavier inputs (chemical fertilizer, manure, pesticide) was added to the orchards during the early period of orchard establishment to stimulate tree growth, which would induce more C emitted to the atmosphere through the practices.

The partitioning of photosynthate among metabolic activities, short-lived tissues and long residence woody organs in fruit trees, is an important process for C sequestration, especially quantifying the allocation to fine roots and investigating its residence time in soils [32]. It was reported that the proportion of photosynthate allocated for fine root construction could account for 30–50% of total global terrestrial primary productivity [33], [34], [35]. *Guo et al*. [36] suggested that the most important channel for fixed C into soils is fine roots because of their short life spans and the quick decomposition of dead roots. Whether the dynamics of fine roots in orchards has such an important role in C sequestration is still controversial, as a considerable portion of photosynthate should be used to support fruit production which is one of the main goals for fruit plantation. In this study, the minirhizotron technique was used to monitor the dynamics of fine roots in order to determine fine root turnover time much accurately. The image analysis showed that the annual fine root turnover index of apple trees is much high, reaching almost 7 for the 5-year-old tree although fine root biomass only accounts for a small percentage of total biomass (<0.1%). The fine roots may not make such a large contribution to C sequestration potential in apple orchards.

The preliminary results from this study showed that the proportion of C emission from the orchard management practices far exceeds the portion of C stocks from both the short-lived tissues and long residence woody tissues in the 5-year-old orchard. When the orchard enters the aging process, the proportion of short-lived tissues is declining. These conclusions were made from only one experiment in three orchards for one growing season. We caution direct interpretation to other geographic sites where the environmental conditions are significantly different. In order to estimate C sequestration potential from apple orchards in China accurately, more long-term field monitoring experiments at different ages are necessary.

Due to the lack of data on soil organic C pool in apple orchards at a provincial/national level, soil organic C sequestration in the apple orchards was not included in the net C sink in this study. Continuous applications of organic fertilizer to agroecosystems would result in the accumulation of organic C in cultivated soils, which could have big potential for C sequestration [37]. As a managed system, the production of apple orchards in China will increase the application of organic fertilizers to replace chemical fertilizers in order to produce high quality fruits and ensure the sustainability of the system. C sequestration potential covering trees, management practices and soils in apple orchards could give more accurate estimation in the future.

It was reported that C sequestration potential in Chinese forests was 5.9×10^3^ Tg C in 2000, equivalent to 41 t C.ha^−1^ [38]. C sequestration from the apple orchards in 2000 was 332.2 Tg C from this study, which is about 5.6% of forest C sequestration. It was estimated that a mean net C sequestration rate in the terrestrial ecosystems which orchard systems were not explicitly included in China was in the range of 190–260 Tg C yr^−1^ during the 1980s and 1990 [39]. Our results indicated that the net sink from the apple orchards in 1990 was 14.1 Tg C, which equals to 4.5% of the reported total C sequestration from the terrestrial systems in China. The Net C sink has gradually increased since 1990 and reached 27 Tg C in 2010. The estimation from this study suggested that 1.6–3.0% of China’s CO_2_ emissions from burning fossil fuels in 2000 [40], [41] could be compensated by C accumulation in apple orchards. Therefore, C sequestration in apple orchards can help to offset industrial CO_2_ emissions.
